# Streamlining regulation of clinical trials – update on the Academy of Medical Sciences review and government’s response

**DOI:** 10.1186/1745-6215-12-S1-A35

**Published:** 2011-12-13

**Authors:** Gary A Ford

**Affiliations:** 1 Newcastle University, NE1 4LP, UK

## 

The UK has a world leading reputation for clinical research. However this position is being undermined by an overly complex regulatory and governance environment that has had a very negative effect on undertaking clinical trials in an efficient way. The European Clinical Trials Directive (EUCTD) was implemented into UK law in May 2004 and was intended to provide greater protection to patients and volunteers participating in clinical trials and increase the quality of trial conduct. At the same time the Research Governance Framework (RGF) was introduced into the NHS. Increasingly the research community has viewed implementation of both the EUCTD and RGF as being disproportionate and ‘gold plated’.

In 2010 the Academy of Medical Sciences was invited by Government to review the regulation and governance of health research and make recommendations to improve current processes. Despite recent attempts to improve parts of the regulation pathway, the review identified that significant challenges remain, including: delays and duplication in obtaining NHS research permissions; a lack of proportionality in the regulation of clinical trials; and a healthcare culture that fails to fully support the value and benefits of health research. The Academy’s report recommended the creation of a new health regulator to rationalise the regulation and governance of health research; steps to streamline the approval of research studies by NHS Trusts; and changes to improve the UK environment for clinical trials.

Since the publication of the Academy’s report a number of positive changes have been made. The 2011 Plan for Growth outlined a package of government measures to foster growth in the healthcare and life sciences and took forward a number of recommendations from the Academy report, including establishment of a new Health Research Authority to streamline regulation and improve the cost effectiveness of clinical trials, and making future funding by the National Institute for Health Research to NHS Trusts conditional on meeting benchmarks. The Government has created a new duty for clinical commissioning groups to promote research innovation and for the NHS Commissioning Board to ensure provision of treatment costs for patients who are taking part in research. The MHRA are introducing a risk based approach to management of clinical trials based on the marketing status of an investigational medicinal produce and the standard medical care that would facilitate a proportionate approach to trial activities. The ongoing review of the EUCTD may offer a further opportunity to reduce the regulatory burden on academic trials.

The above changes are welcome and should result in reductions in the time to obtain approvals and permissions for clinical trials and some reduction in trial management costs. However it remains to be seen whether the health research regulatory agency will have sufficient authority to achieve more efficient working between regulators, researchers and the NHS to maintain the UKs historical position as a world leader in designing and delivering high quality clinical trials.

